# Supplementary material for: Patterns of Intron Gain and Loss in Fungi
Source: PLoS Biol. 2004 Nov 30;2(12):e422. doi: 10.1371/journal.pbio.0020422 (PMC532390; doi:10.1371/journal.pbio.0020422)
Supplement: Table S1 — Also available at http://genes.mit.edu/NielsenEtAl/. (4.3 MB ZIP). [file pbio.0020422.st001.zip › NielsenEtAl/html/1331.html]

AN4096.1.NCU06407.1.MG06243.1.FG05503.1


```
 CLUSTAL W (1.82) Multiple Sequence Alignments - Introns Inserted


Sequence 1: NCU06407.1	1131 aa
Sequence 2: FG05503.1	1073 aa
Sequence 3: MG06243.1	1114 aa
Sequence 4: AN4096.1	988 aa
Alignment Length: 1177 aa
Number Identitical Residues: 319 aa
Alignment Score (without introns) 17535


MG06243.1 	---MSSSQPTTEVQTFATLDLDTEAQLMRADGVDLNGFQLDGTPNGSLHDDAPPQNVMDS
NCU06407.1	MPPADSQALNNNNNSLSSVDLN-----FAPAGGDTHGHPPHSDPAA---FQTHPLDKLDT
FG05503.1 	---MSGRAVLKPQHAPSPFDS----------APQLYRDVPGLHPAP---SPSHG-ALMDT
AN4096.1  	------------------------------------------------------------
          	                                                            

MG06243.1 	S----------------HFGDFQFPFQTINDESTIVAMTDASPPVDVFPPTQHRPSISSI
NCU06407.1	SNDALGSIDAAHDLVDGHYADFQFHYDDDLDGSPMTGLGSATFDDHAPDVFSTTPHAPSM
FG05503.1 	S----------------HFDDFSFAYQGLPDQSPLVSLADHAHASQPPTPF---PQHQAM
AN4096.1  	----------------------MNPFKPDESSSAHSALPLAPGHDDVALPDDFDSNSPAS
          	                         :.   . *.  .:   .   .     .  .   : 

MG06243.1 	PADRRGSIASTTAQAPSYNGNRASQRRPSQVTSEDGSPDSNGNGLLLEDPGADEFGLISS
NCU06407.1	DS---ITVQTSTYNNNGANTGNGGQRQQSMDMSESNTPDSNQNAGNSLEETVDEFGLATA
FG05503.1 	SG---LSHAGLPFGALPP-----GNRSQSIEGSETGQGRISPSSNALEDSNADEFGLASR
AN4096.1  	SR-------------------------------EPGSAQTGGDAAGFQDGNRDN----AA
          	                                 * .    . ..    :   *:    : 

MG06243.1 	GPGDASGAG-KFKDDKGESMPAWSELKTKAGKERKRLPLACIACRRKKIRCSGEKPACKH
NCU06407.1	SSVDGSAP--KGKGDKTDNTPAWSELKTKAGKERKRLPLACIACRRKKIRCSGEKPACKH
FG05503.1 	NRADGTDLGGKHKEDKADATPAWSELKTKAGKERKRLPLACIACRRKKIRCSGEKPACKH
AN4096.1  	DRGDDTPQT---HQEQAGDKPPWSETKTKAGKERKRLPLACIACRRKKIRCSGEKPACKH
          	.  * :      : ::    *.*** **********************************

MG06243.1 	CLRSRIPCVYKVTARKAAPRTDYMAMLDKRLKRMEERIIKTVPKSEQDGIAATVMRSVLK
NCU06407.1	CLRSRIPCVYKVTARKAAPRTDYMAMLDKRLKRMEERIIKIVPKSEQE-GASTVTRAVVK
FG05503.1 	CLRSRIPCVYKVTTRKAAPRTDYMAMLDKRLKRMEERIIKVIPKSDQE-VASSVTRAVVK
AN4096.1  	CSRSRIPCVYKVTTRKAAPRTDYMAMLDKRLKRMEDRVIKTIPKDEFR-DMGAIGRANVR
          	* ***********:*********************:*:** :**.:     .:: *: ::

MG06243.1 	PAIPGTLSASKAAAKKRGADEAFGPELDNWARSSTKSKTDTSGRPATLALHEAEENKLLQ
NCU06407.1	PAIPGTLNNMKQANKKRGADEAFGSDLENWARGPAKTKIDAPNRTATIHIQETEESKLLL
FG05503.1 	PAIPGTVP-SNKSTKKRGAEEAFGPDLEAWAKAPSKPKIDGDDRPSSLQVQETEENKLQH
AN4096.1  	PAQPGQAV---KNQKKRSADEAFAAELEQWARG---SRSGPQDTFPMRREGKPDGPSLMT
          	** **         ***.*:***..:*: **:.   .: .  .  .     :.:  .*  

MG06243.1 	EGADALPSREIQEHLAEVFFDDIYGQAYHLLHRPSYMRKLR2AGTLPPVLILSVCAVSAR
NCU06407.1	EGGDALPSKEIQEHLAEVYFENIYGQAYHLLHKPSFMRKLK2AGALPPVLILSVCAIAAR
FG05503.1 	EGTDALPSKEIQEHLAEVFFDNVYGQSYHLLHKPSYMRKLK2NGTLPPVLILTVCAVAAR
AN4096.1  	EGAEFLPSLEIQEHLAEVFFDCVYGQSYLLLHKPSFVRRLK~ARTVPPVLILAVCAVSAR
          	** : *** *********:*: :***:* ***:**::*:*:   ::******:***::**

MG06243.1 	FSTHPKLNTN-PNFLRGEEWASHARDIVMRRYEWPNITILTCLLILGLHEFGTCHGGRSW
NCU06407.1	FSHHPKLNSS-PNFLRGEEWAAAARDIVTKRYEWPNITILTCLLILGLHEFGTCHGGRSW
FG05503.1 	FTSNPLVSSSGPEFLRGEEWATHAREICTRRYEWPNLTILTCLLILGLHEFGTCQGGRSW
AN4096.1  	FSTHPQINSE-PPFLRGENWASPAATIALKRHDEPNITILTVFLLLGLHEFGTCHGGRSW
          	*: :* :.:. * *****:**: *  *  :*:: **:**** :*:*********:*****

MG06243.1 	ALGGQAIRMAFALQLHKDLEYDP-----HSGRAQLSFIDREIRRRTMWACFLMDRFNSSG
NCU06407.1	SLGGQAIRMAFALQLHKDLEHDP-VR--QAGNTQLSFIDREIRRRTMWACFLMDRFNSSG
FG05503.1 	ALGGQAIRMAFALQLHKDLEYDPSGR--NGAKKQLSFIDREIRRRIMWACFLMDRFNSSG
AN4096.1  	SFGGQAMRMAYALQLHRELDYDPLLSQGNGGGVQLSFTDREIRRRTMWACFLMDRYNSSG
          	::****:***:*****::*::**   ..:..  **** ******* *********:****

MG06243.1 	TERPTFIKEETVKIPLPVKEKYFQLDIPGATEDLNGPTLNTMDLDEDQATQFKENMGVAA
NCU06407.1	TDRPTFIREETLRVPLPIKEKNFQYDMPGPTETLSGEVLGPLLE-DQDASMARDNLGVAA
FG05503.1 	TDRPMFVREDTIQIPLPVKEKYFQFDMPAPTEMLDGQIPHPASPNDGQLADARENMGVAA
AN4096.1  	SQRPPIGNEKFIQIQLPIKESHFQMEIPGPTEDLEGNVLNPVPEDVGQLSNAKSNMGVLA
          	::** : .*. ::: **:**. ** ::*..** *.*    .   .  : :  :.*:** *

MG06243.1 	YMVRSIAIWGRIINYLNQGGKELDPHPMWSPDAEYAKLIKQSEDLPASLPDSLQYNRENL
NCU06407.1	WMIKAISLWGRIINYLNQGGRELDPHPMWSPESGYAKLIKQTEDILTDLPDSLLYTPDNL
FG05503.1 	YLVRAIALWGRIITYLSQGGKELDPNPMWEDESQYIKHLNDVVNLEASLPLSLRYSVENL
AN4096.1  	YIIRAVVIWGRIVDYLNLGGKRRDNHPLWSPESGYSRLRKQIEDFSASLPSHLHFTYENL
          	:::::: :****: **. **:. * :*:*. :: * :  ::  :: :.**  * :. :**

MG06243.1 	KLHDTEGMANQFLFLHISIQQNILFMNRFAVS-SPGSITQQDVPKSFVTKAGAKAFAAAN
NCU06407.1	HLHETDNMANQFLFLHIAIQQNILFMNRFAVS-SPRGQPDQEIPKAFVTKAGAKAFAAAN
FG05503.1 	EVHKTENTAGQFLFMHICLQHNILFVSRAAMS-ARK---QQGAHNDFFSEASKRTFNAAN
AN4096.1  	QVHAADRVANQFLFLHIIIHQNTLFLNQFAIPLSPGGRPPRDMPRTFLSNAGRAAVEAAH
          	.:* ::  *.****:** :::* **:.: *:. :  . . :   . *.::*.  :. **:

MG06243.1 	RMSELLKDAEP--YLVSAPFVGYCTFLSSTVHIFGIFSGNPAIESTSKRNLATNVRFLSK
NCU06407.1	RVSELLKDAES--HFVTAPFTGYCAFLSSTVHIFGIFSGNPTLEATSKRNLATNVKFLSK
FG05503.1 	RISELLREAEQPRCFVSAPFAGYCAFSSTTVHILGIISLNPSMKPAAEANLATNVKYLTK
AN4096.1  	HISALINHAAP--YPLTVPFAGYCAYSASTVHIWGIFSKNTQLEARSKENLRHTYRYLNK
          	::* *:..*      ::.**.***:: ::**** **:* *. ::. :: **  . ::*.*

MG06243.1 	MKRFWGMFHFMTENLREQYRACADAARQGNPANEN-NSPQIFQYGDWFDRYPHGVSQSDF
NCU06407.1	MKKYWGMFHWMSENLREQYRTCADAARQGTPANE--NPTSIFQYGDWFDRYPHGVSQSDF
FG05503.1 	MKKYWGMFHWMVENIRTQYRNALDAVRAGENVNERATQSSYLQYGDWFNRYPHGLSDAEF
AN4096.1  	MKKYWGMFHYMVESAKDRYRQFADAALKGTVASQNGPVAPMFQYGDWFDKYPHGVSRQHW
          	**::*****:* *. : :**   **.  *  ..: .  .  :******::****:*  .:

MG06243.1 	LDPVKYRAKEKGNDAVLEQKPELATVEEFMTKMSPRS----------TEGAVNNGHAGKP
NCU06407.1	LDPAIYKKKEKGDDAVLEQKPELHTVEEFFTTLSPQAGGGDGGSVNGSTASRQQQQAQQQ
FG05503.1 	MDPATHKRKDLGADGVLEAKPELQSVEEYFSTLP-------------TPQSIEHKDSIRA
AN4096.1  	ED-SSHAARQKDDEAVMSQKPDLQSVEDFFASLS-------------PTPQPNDSRRKHS
          	 *   :  :: . :.*:. **:* :**::::.:.             .    :.    : 

MG06243.1 	QPAKRKSIAKRGGSTTSRSEHQ---PDPIMTDVGKHGATEQQAQHQPPQQGNSRRRFSGS
NCU06407.1	QQLKRKAMTRKISGLPQQNHPNGPDPIQAEFSPHQQQHPPPTAEQIHAARLQHQRNFSGA
FG05503.1 	AAPKRKQGNKKQTGMSVQSG--------------QPLDPMQGTDTDSVSVGGQERRFSGA
AN4096.1  	RAGSRIDLTTMDQTTPTPTS---------------QQIVDVNLETSPGILGTSGTGFPQP
          	   .*          .  .                        :            *. .

MG06243.1 	MGG--QTN--APSFSPLTIPQSANQAPYHTLSPISPVAMSAFPHHNHHHQNSQFYSPELL
NCU06407.1	GPSPIQTSGPPGSFNSLSVQAQQQAHFS-PVSPVG-VSHAHLSHH----HPGSFFPPDPF
FG05503.1 	LG--LQTTGTAG-FNPLAVSNPQNSAFSTAMSPMSPANMTAFSHHG---HTPTFFPPELL
AN4096.1  	MFPQNRGHQTFG-HSPFDFTIPVTDQLPQLDRQFVYDSFGGFKPS------TNSYVPNPN
          	     :   .   ...: .              .       :            : *:  

MG06243.1 	SLSLSQQT-TAGILPQLDRQLVFGAYGGLDPGMTSQMLDGGGIWDGMG-GQRHGDGGRRT
NCU06407.1	SLPAHLGAAHAHGMSHLDRQLVFGAYG---GPTSAGLHHEPGLVGGGGGGSGGGGAGGWG
FG05503.1 	AM--NFGSGSNTNIDPLDRQLVFGGYS---VDVGTGLGSGHDMMTGMGWDAVPGTQSDED
AN4096.1  	DQPPSFSTMNGADVAPPP------------GPQDTGLPNQDIFTGQMDPNAPSGAGEYYQ
          	  .    :     :                    : :     :    . .   *      

MG06243.1 	HTSTAEVLHAALVGHTLTHHNHPGHHAHEGASSAWFMPFNMEPPEIGHDLT---------
NCU06407.1	GGHDSENGRDGPDGLHAAFSN--------EPSSAWFMPFNMEPPEIGGDTLGGLGGALGG
FG05503.1 	LQSRRSNMKAGMNGQGAGMADGA-GLSGPEASSAWFMPFNMEPPEMGQDPSFNMG-----
AN4096.1  	PSAWFLPFNLDPIGAGVSLDPGS-QPDAGGDLSGFGATGNMPMGAYDLGMS---------
          	        .    *        .         *.:  . **     . .           

MG06243.1 	-SLDGLGAMFGGA~MTTQGGMGSGGGGGNPNHNGH----
NCU06407.1	GGLDSFAGMFGVG~AGGGGGGGGGGGMGGMHHGH-----
FG05503.1 	-GIDPFTGVFGT-~--GGSGLATPNAMGGLQQQGP----
AN4096.1  	-GMDHNHGSQMKG2PAIDLGGSALTSILDVEIAQLLLVA
          	 .:*   .    .      * .   .  . .       :
```
